# Analysis of spatiotemporal fidelity in quantitative 3D first-pass perfusion cardiovascular magnetic resonance

**DOI:** 10.1186/s12968-017-0324-z

**Published:** 2017-01-27

**Authors:** Lukas Wissmann, Alexander Gotschy, Claudio Santelli, Kerem Can Tezcan, Sandra Hamada, Robert Manka, Sebastian Kozerke

**Affiliations:** 1 Institute for Biomedical Engineering, University and ETH Zurich, Gloriastrasse 35, 8092 Zurich, Switzerland; 2 Department of Cardiology, University Hospital Zurich, Zurich, Switzerland; 3 Division of Internal Medicine, University Hospital Zurich, Zurich, Switzerland; 4 Department of Cardiology, RWTH Aachen University, Aachen, Germany; 5 Institute of Diagnostic and Interventional Radiology, University Hospital Zurich, Zurich, Switzerland; 6 Division of Imaging Sciences, King’s College London, London, UK

**Keywords:** First-pass myocardial perfusion, Myocardial blood flow, Modulation transfer function, *k-t* PCA, *k-t* SPARSE-SENSE, 3D-MTF, Whole-heart perfusion

## Abstract

**Background:**

Whole-heart first-pass perfusion cardiovascular magnetic resonance (CMR) relies on highly accelerated image acquisition. The influence of undersampling on myocardial blood flow (MBF) quantification has not been systematically investigated yet. In the present work, the effect of spatiotemporal scan acceleration on image reconstruction accuracy and MBF error was studied using a numerical phantom and validated in-vivo.

**Methods:**

Up to 10-fold scan acceleration using *k-t* PCA and *k-t* SPARSE-SENSE was simulated using the MRXCAT CMR numerical phantom framework. Image reconstruction results were compared to ground truth data in the *k-f* domain by means of modulation transfer function (MTF) analysis. In the *x-t* domain, errors pertaining to specific features of signal intensity-time curves and MBF values derived using Fermi model deconvolution were analysed. In-vivo first-pass CMR data were acquired in ten healthy volunteers using a dual-sequence approach assessing the arterial input function (AIF) and myocardial enhancement. 10x accelerated 3D *k-t* PCA and *k-t* SPARSE-SENSE were compared and related to non-accelerated 2D reference images.

**Results:**

MTF analysis revealed good recovery of data upon *k-t* PCA reconstruction at 10x undersampling with some attenuation of higher temporal frequencies. For 10x *k-t* SPARSE-SENSE the MTF was found to decrease to zero at high spatial frequencies for all temporal frequencies indicating a loss in spatial resolution. Signal intensity-time curve errors were most prominent in AIFs from 10x *k-t* PCA, thereby emphasizing the need for separate AIF acquisition using a dual-sequence approach. These findings were confirmed by MBF estimation based on AIFs from fully sampled and undersampled simulations. Average in-vivo MBF estimates were in good agreement between both accelerated and the fully sampled methods. Intra-volunteer MBF variation for fully sampled 2D scans was lower compared to 10x *k-t* PCA and *k-t* SPARSE-SENSE data.

**Conclusion:**

Quantification of highly undersampled 3D first-pass perfusion CMR yields accurate MBF estimates provided the AIF is obtained using fully sampled or moderately undersampled scans as part of a dual-sequence approach. However, relative to fully sampled 2D perfusion imaging, intra-volunteer variation is increased using 3D approaches prompting for further developments.

## Background

Diagnosis of ischemia in patients with known or suspected coronary artery disease (CAD) is increasingly being performed using cardiovascular magnetic resonance (CMR) first-pass perfusion imaging. Perfusion CMR outperforms other imaging techniques such as positron emission tomography (PET) and single photon emission computed tomography (SPECT) in terms of spatial resolution and operates without ionizing radiation. Compared to coronary angiography and the assessment of fractional flow reserve, perfusion CMR is non-invasive and has been proven suitable for patients with intermediate probability of significant CAD [1]. Numerous authors have compared the diagnostic performance of perfusion CMR to SPECT [2–6] and PET [7–10], and found that CMR performs at least comparably to these methods [11]. Studies comparing perfusion CMR to stress echocardiography and perfusion computed tomography report similar results [12–14].

Detection of small ischemic regions such as sub-endocardial perfusion defects is enabled by the high spatial resolution offered by CMR [15]. In addition to sufficient spatial resolution, whole-heart coverage is desired to accurately assess the size and extent of perfusion deficits [16]. These demands have triggered the development of three-dimensional (3D) scanning techniques employing advanced undersampling strategies for efficient data acquisition [17–19]. The importance of whole-heart imaging has further been stressed by authors evaluating the volumetric ischemic burden as a marker of significant CAD [20–22]. Optimization of scanning efficiency has also been made in the temporal domain. Multiple authors have proposed perfusion CMR throughout the cardiac cycle to assess differences in perfusion between heart phases and to combine cine and perfusion imaging into a single scan [23–25]. Alternatively, interleaved acquisition at different heart phases may be used to separately capture blood pool and myocardial enhancement for improved perfusion quantification [26, 27] or frame-by-frame T1 mapping [28].

Absolute quantification of perfusion CMR has gained significant attention in the past decade since clinical advantages have been pointed out [29, 30]. Several technical aspects of myocardial blood flow (MBF) estimation from reconstructed images have been investigated mostly using single or multi-slice 2D imaging. Special focus has been put on the development of mathematical models for MBF estimation [31–34] and comparison between them [35, 36]. Zarinabad et al. have compared voxel-wise vs. spatially averaged (sector-wise) estimation of MBF [36] and highlighted the importance of accurate bolus arrival time estimation [37]. Most recently, the feasibility of 3D CMR perfusion quantification has been demonstrated [25, 27].

Perceived image quality and deviation from a fully sampled reference image have traditionally been used as direct measures to validate spatiotemporal scan acceleration methodology [38–40]. A more general approach is the use of the modulation transfer function (MTF) concept to characterize the ability of a MR system to correctly capture spatial and temporal frequencies [41]. A perturbation of the system combined with linear regression is used yielding the MTF derived from the slope, and an artefact map based on the ratio of slope and intercept of the linear fit. This method is well suited for linear reconstruction methods, but application to non-linear reconstruction techniques such as compressed sensing is also feasible if linearization about a suitable expansion point is used. Consequently, the MTF approach can be employed to compare spatiotemporal performance of linear and non-linear reconstruction algorithms, such as *k-t* PCA and *k-t* SPARSE-SENSE [42, 43].

The present study introduces a linearized MTF approach to evaluate *k-t* PCA and *k-t* SPARSE-SENSE in the context of highly accelerated, fully quantitative 3D myocardial perfusion imaging. MTF maps derived from numerical phantoms and in-vivo data are used to investigate changes of spatiotemporal fidelity introduced by undersampling. Furthermore, errors in signal intensity-time curves are analysed and their influence on MBF estimation is highlighted. MRXCAT simulation of a sub-endocardial lesion reveals the ability of the proposed methodology to identify small ischemic territories. Finally, simulation results are validated in-vivo comparing 3D *k-t* PCA, 3D *k-t* SPARSE-SENSE and fully sampled 2D imaging.

## Theory

### k-t PCA and k-t SPARSE-SENSE

*k-t* PCA and *k-t* SPARSE-SENSE are reconstruction methods based on differing principles both suited for highly accelerated MRI.

In *k-t* PCA, data is acquired on a Cartesian grid, which is shifted in *k*-space for each time frame as in *k-t* SENSE [38]. The centre of *k-*space is fully sampled in all time frames, providing an image series with low spatial and high temporal resolution termed as training data. Before further processing, the data matrix **D** originally acquired in *k-t* space is Fourier transformed to the *x-f* domain,

1 $$ \mathbf{P}={\mathrm{F}}_{k- t\to x- f}\mathbf{D}. $$

F_*k-t*→*x-f*_ denotes the Fourier transform from *k-t* to *x-f* space. The training data are used to determine the temporal principal components (PCs) of the dataset by transforming data from *x-f* space to *x-pc* space using principal component analysis (PCA),

2 $$ \mathbf{P}=\mathbf{W}\mathbf{B}, $$

where **P** and **W** are matrices representing the data in *x-f* and *x-pc* space, respectively, and matrix **B** contains the PCs. By assuming spatial invariance of the PCs, the same PCs can be used to unfold the aliased data. The reconstruction problem can then be solved via [42]

3 $$ {\mathbf{w}}_x={\mathbf{M}}^2{\mathbf{E}}^H{\left(\mathbf{E}{\mathbf{M}}^2{\mathbf{E}}^H+\lambda \boldsymbol{\Psi} \right)}^{\dagger }{\mathbf{p}}_{\mathrm{alias}, x}, $$

where **w**_*x*_ and **p**_*x*_ are vectors representing the rows of **W** and **P** at position *x.* **w**_*x*_ contains the weights of the aliased voxels in **p**_alias,*x*_, **M** ^2^ is the signal covariance, **Ψ** indicates the noise variance, and **E** is the encoding matrix. The dagger represents the Moore-Penrose pseudoinverse and superscript *H* the conjugate transpose. The reconstructed image **i**_*x-f*_ in *x-f* space is obtained using

4 $$ {\mathbf{i}}_{x- f}={{\mathbf{B}}_x}^{\dagger }{\mathbf{w}}_x $$

followed by Fourier transformation to the *x-t* domain.

In contrast to *k-t* PCA, data in *k-t* SPARSE-SENSE are pseudo-randomly undersampled with a higher sampling density near the *k-*space centre decreasing towards the edge [44]. The reconstruction problem reads

5 $$ \underset{\mathbf{i}}{ \arg \min }{\parallel \mathbf{d}-\mathbf{Ei}\parallel}_2^2+\lambda {\parallel \Phi \mathbf{i}\parallel}_1, $$

with the encoding matrix **E** as above, the data **d** expressed as a vectorised form of **D**, and **i** the image to be reconstructed. Φ represents a sparsifying transform and *λ* is the regularization parameter. In *k-t* SPARSE-SENSE, the reconstruction equation is minimized using a POCS-like algorithm alternating between data consistency and soft-thresholding [45, 46] leaving the acquired data unchanged, or non-linear conjugate gradient optimization [44]. Common choices for Φ include the temporal Fourier transform (FT), temporal PCA or a mixture of both starting with the temporal FT for the first iterations, followed by PCA for the remaining iterations [47].

### Spatiotemporal modulation transfer functions

Traditionally, modulation transfer functions (MTF) are used to describe an imaging system’s ability to portray an object. Chao et al. [41] have adopted the concept for the evaluation of accelerated MRI. The relationship between the object **ρ** and its image **i** can be formulated as

6 $$ \mathbf{i}=\mathbf{H}\boldsymbol{\uprho } +\mathbf{n}, $$

with the modulation transfer function **H** and noise **n**. Explicit calculation of **H** for large imaging problems, such as dynamic 3D imaging, can be infeasible or computationally too expensive. To address this issue, a perturbation approach

7 $$ {\mathbf{i}}_{\boldsymbol{\upxi}}={\mathbf{H}}_B\left(\boldsymbol{\uprho} +\boldsymbol{\upxi} \right)+{\mathbf{h}}_A={\mathbf{H}}_B{\boldsymbol{\uprho}}_{\boldsymbol{\upxi}}+{\mathbf{h}}_A $$

can be used. A small perturbation **ξ** is added repeatedly to the object **ρ. ρ**_**ξ**_ and **i**_**ξ**_ are the perturbed object and image, respectively. **H**_*B*_ and **h**_*A*_ are analogous to the MTF and noise in eq. (6). Multiple realizations of eq. (7) with different perturbations can be solved for **h**_*A*_ and **H**_*B*_ using linear regression, which results in slope **H**_*B*_ and intercept **h**_*A*_. This MTF formalism can be applied to study the effects of scan acceleration. To this end, the image **i**_*R*=1_ reconstructed from fully sampled data is used as the true object and the reconstructed image **i**_*R*>1_ from undersampled data is its imaged version. The adapted version of eq. (7) reads

8 $$ {\mathbf{i}}_{R>1}={\mathbf{H}}_B{\mathbf{i}}_{R=1}+{\mathbf{h}}_A. $$

Instead of the 2D MTF [41], a 3D MTF **H**_*B*_(*k*_*y*_ *,k*_*z*_ *,f*) portraying two spatial and the temporal frequency directions is necessary for Cartesian dynamic 3D imaging. The frequency encoding direction *k*_*x*_ can be omitted, or used for averaging, since no undersampling is applied and thus the MTF is constant along this direction. The MTF can be computed as [41]

9 $$ \mathbf{M}\mathbf{T}\mathbf{F}\left({k}_y,{k}_z, f\right)=\sqrt{\frac{\sum_x{\left|{\mathbf{H}}_B\left( x,{k}_y,{k}_z, f\right)\right|}^2}{N_x}}, $$

where *N*_*x*_ is the number of readout profiles. Similarly, the signal-to-artefact map (S2A) can be derived relating the MTF to the intercept of the linear regression and to the object itself:

10 $$ \mathbf{S}\mathbf{2}\mathbf{A}\left({k}_y,{k}_z, f\right)=\mathbf{M}\mathbf{T}\mathbf{F}\left({k}_y,{k}_z, f\right)\cdot \sqrt{\frac{\sum_x{\left|\boldsymbol{\uprho} \left( x,{k}_y,{k}_z, f\right)\right|}^2}{\sum_x{\left|{\mathbf{h}}_A\left( x,{k}_y,{k}_z, f\right)\right|}^2}}. $$

While the formalism is directly valid for the linear *k-t* PCA in eq. (3), for *k-t* SPARSE-SENSE (eq. (5)) an approximately linear relationship between fully sampled and accelerated imaging is assumed based on linearization about a suitable expansion point. This expansion point corresponds to the magnitude of the unperturbed object at each position in *k*_*y*_ *-k*_*z*_ *-f* space.

In the original interpretation of the MTF formalism a true object and its imaged version are compared. The natural upper bound for the MTF is 1, indicating that a certain voxel in *k-f* space perfectly reproduces the corresponding object part. Lower values of the MTF indicate image degradation by the imaging system. Note that this strict physical constraint not necessarily applies to scan acceleration. Especially at the *k-f*-space edges, where the signal-to-noise ratio (SNR) is low, the effect of undersampling and subsequent reconstruction might also increase *k-f* space magnitudes, resulting in MTF values above 1. Therefore, only the central *k-f*-space parts of the MTF should be evaluated.

### Myocardial blood flow quantification

There are a number of methods estimating myocardial blood flow (MBF) from first-pass perfusion CMR. The most direct approach is to derive MBF estimates from the relationship between contrast agent concentrations at the inlet *c*_AIF_(*t*), referred to as arterial input function (AIF), and in the myocardial tissue *c*_MYO_(*t*), using [48].

11 $$ {c}_{\mathrm{MYO}}(t)={R}_F(t)\otimes {c}_{\mathrm{AIF}}(t). $$

The flow-weighted impulse response function *R*_*F*_ = *F · R*(*t*) comprises the MBF estimate *F* and a normalized, decaying function *R,* with *R*_*F*_(*t* = 0) = *F*. The impulse response function can either be explicitly computed by model-free deconvolution, or approximated using a suitable mathematical representation. The most common choice for *R*_*F*_ is approximation using the 3-parameter Fermi model [27, 31],

12 $$ {R}_F(t) = F\cdotp \frac{1+\beta}{1+\beta \cdotp {e}^{\alpha t}}. $$

In this equation, *F* is the MBF estimate, and *α*, *β* are further fitting parameters. Note that the units of measurement for *c*_AIF_(*t*) and *c*_MYO_(*t*) are mmol/mL, while the amount of contrast agent in the myocardium measured by indicator dilution theory is in units of mmol/g of tissue. This discrepancy is implicitly corrected by scaling *F* by the myocardial tissue density of 1.05 g/mL [49].

## Methods

### In-vivo measurements

In-vivo CMR experiments were performed in 10 healthy volunteers (4 males) on a Philips Achieva 1.5 T scanner (Philips Healthcare, Best, The Netherlands) using a 5-channel cardiac coil array. Volunteers had an average age of 26.2 ± 4.7 years and underwent CMR upon written informed consent in accordance with ethics regulations approved by the local ethics committee. Dynamic contrast enhanced CMR was conducted twice per volunteer and at least 20 min apart. Gadobutrol (Gadovist, Bayer Schering Pharma, Germany) at 0.075 mmol/kg b.w. dose was injected as contrast agent, followed by a 30 mL saline flush at 4 mL/s. Volunteers were measured during instructed breath-holding.

A saturation-recovery dual-sequence spoiled gradient echo sequence with ECG-triggering was used to acquire one image pair per heartbeat. The interleaved acquisitions [50] consisted of a 2D aortic scan for arterial input function (AIF) assessment and an end-systolic left-ventricular scan to capture myocardial enhancement, as proposed earlier [27]. Myocardial enhancement was assessed using 3D imaging accelerated by *k-t* PCA (*N* = 7 measurements), *k-t* SPARSE-SENSE (*N* = 7), and fully sampled single-slice 2D imaging (*N* = 6) for comparison. To limit the amount of contrast agent administered and the examination time per volunteer, only two injections per volunteer were carried out. This resulted in three groups of volunteers, allowing comparison of *k-t* PCA or *k-t* SPARSE-SENSE with fully sampled 2D imaging (*N* = 3 for both), and direct inter-comparison between the accelerated sequences (*N* = 4).

**Fig. 1:**
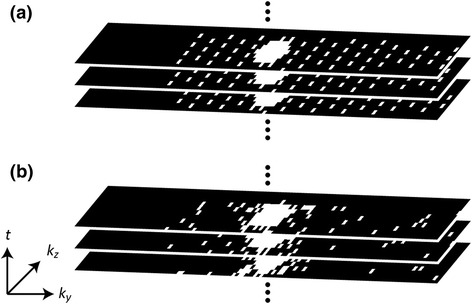
3D *k*-space sampling patterns in *k*_*y*_-*k*_*z*_ for *k-t* PCA (**a**) and *k-t* SPARSE-SENSE (**b**). The regular undersampling pattern in *k-t* PCA is shifted along the temporal dimension using a fixed pattern. In *k-t* SPARSE-SENSE the sampling is random with high sampling probability density in the *k*-space centre decreasing towards the edge. The randomness ensures temporal variability of the sampling pattern. 75% partial Fourier sampling was employed along *k*_*y*_ and *k*_*z*_ in both cases

All myocardial enhancement scans were run with WET saturation preparation [51] using a saturation to acquisition time (*T*_SAT_) of 150 ms. Accelerated 3D imaging parameters were: nominal scan acceleration: 10x, net acceleration factor without partial Fourier: 7.4–7.8, 11×7 training profiles in *k*_*y*_ and *k*_*z*_, spatial resolution: 2.3×2.3×10 mm^3^, 10 contiguous slices, typical field-of-view: 320×320×80 mm^3^, flip angle: 15°, acquisition window: 189–216 ms, *T*_*R*_: 1.89–1.93 ms, *T*_*E*_: 0.74–0.78 ms. 62.5% and 75% partial Fourier sampling was applied in frequency and in both phase encoding directions, respectively. An elliptical *k*-space shutter was used on both the undersampled grid and the training portion. Equal undersampling rates were used in *k-t* PCA and *k-t* SPARSE-SENSE. Examples of sampling patterns as applied in-vivo for both *k-t* methods are illustrated in Fig. 1. Fully sampled 2D myocardial enhancement scans were run with the following parameters: spatial resolution: 2.3×2.3 mm^2^ in-plane, slice thickness: 10 mm, flip angle: 15°, acquisition window: 188–225 ms. *T*_*R*_, *T*_*E*_ and partial Fourier factors were the same as for 3D imaging, resulting in comparable acquisition windows.

2D AIF imaging was planned orthogonally to the ascending aorta in transverse view, with a separate WET saturation preparation pulse. An ultrashort *T*_SAT_ of 3.7 ms was enabled using a central-out profile order, i.e. acquisition started at the *k*-space centre, continued outwards and concluded at the most distant point from the centre. Further 2D scan parameters were: 3x *k-t* PCA acceleration, 11 training profiles, spatial resolution: 3.5×3.5 mm^2^, slice thickness: 10 mm, field-of-view: 260×300 mm^2^, flip angle: 15°, acquisition window: 40–48 ms, *T*_*R*_: 1.67 ms, *T*_*E*_: 0.58 ms.

In addition to contrast-enhanced imaging, baseline *T*_1_ values were measured in all volunteers using modified Look-Locker inversion recovery (MOLLI) imaging [52]. MOLLI acquisitions were done before the first and second contrast administration. Population average pre-contrast myocardial and left-ventricular *T*_1_ values for the first and second injection were determined from these MOLLI *T*_1_ maps. These average *T*_1_ values were subsequently used for signal intensity to contrast agent concentration conversion, as outlined below.

## Image reconstruction

*k-t* PCA and *k-t* SPARSE-SENSE reconstructions were implemented in ReconFrame (Gyrotools LLC, Zurich, Switzerland) and Matlab R2014a (MathWorks, Natick MA, USA). Sensitivity maps were derived from a separately acquired reference scan. The *k-t* SPARSE-SENSE implementation comprised soft thresholding and a combination of temporal FT (10 iterations) and PCA (iteration 11 onwards) as sparsifying transforms [47]. Reconstruction voxel sizes of 2×2 mm^2^ and 1.25×1.25×5 mm^3^ were achieved using zero-filling of the 2D AIF image and the accelerated 3D scan, respectively. All reconstructed in-vivo images were manually segmented to yield regional signal intensity-time curves.

### Modulation transfer function analysis

Numerical simulations were performed to compare images reconstructed from undersampled data with fully sampled references using MTFs. A fully sampled 3D numerical phantom was created using the MRXCAT simulation framework [53]. Phantom parameters were: spatial resolution: 2.3×2.3 mm^2^, slice thickness: 5 mm, 10 slices, *T*_*R*_ */T*_*E*_: 2.0/1.0 ms, flip angle: 15°, contrast agent dose: 0.075 mmol/kg b.w., 5 receive coils, myocardial blood flow (MBF): 1 mL/g/min. 64 noise realizations with equal noise statistics were performed, each comprising 11 different perturbations for 4 different acceleration factors (cf. below). In each realization, 11 identical datasets were generated, which were individually perturbed by multiplication with factors 0.95–1.05 in steps of 0.01, and subsequent degradation by noise (SNR = 20). Scaling was done to ensure that a certain signal intensity range was covered for linear regression analysis. Compared to completely random perturbations without scaling, this approach ensured a spread of signal values at every *k*-space position. This resulted in a drastically reduced number of iterations required to probe linearity at all spatiotemporal frequency positions.

**Fig. 2:**
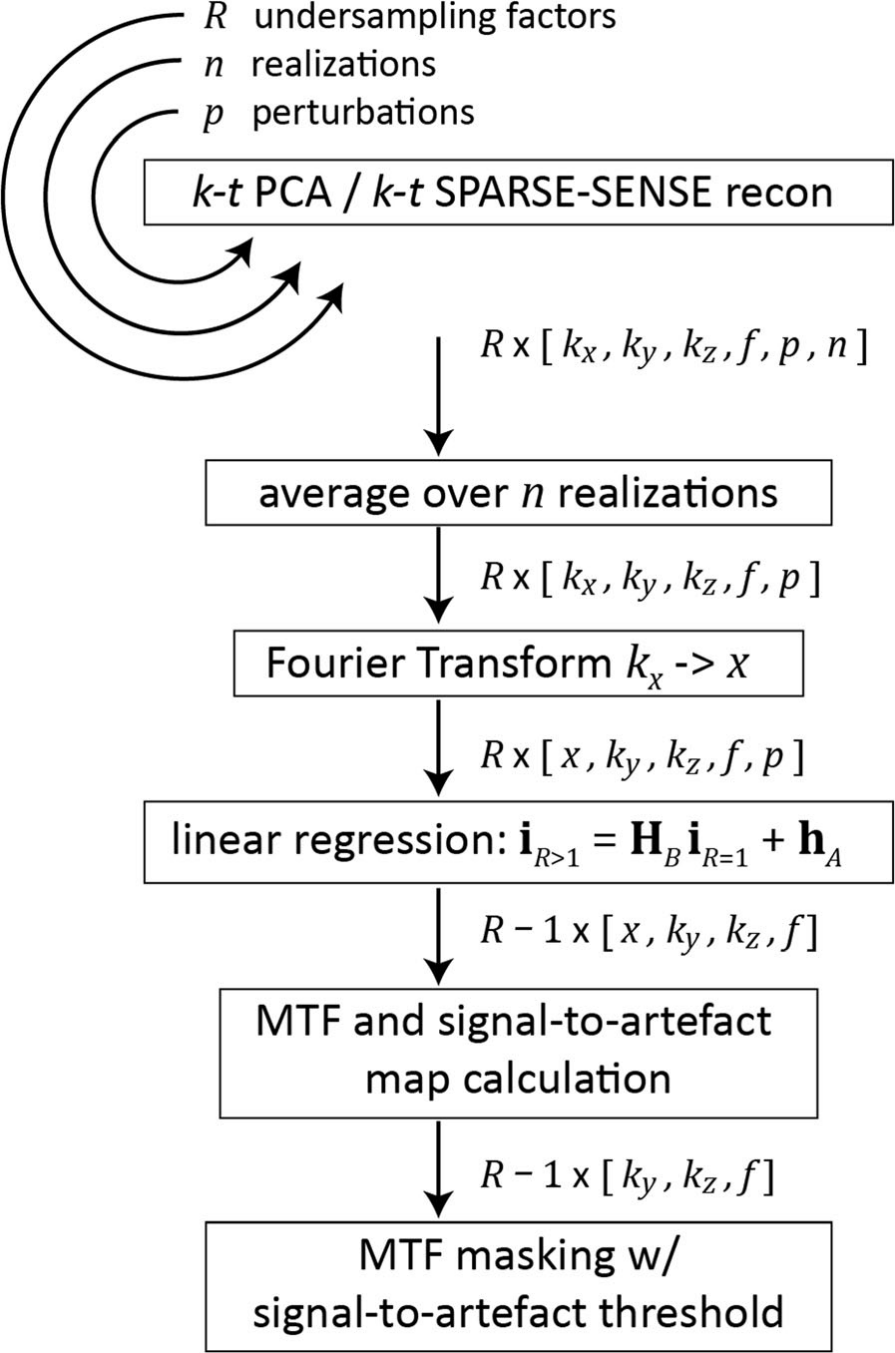
Workflow for the simulation of modulation transfer functions (MTFs). Image reconstruction is repeated for *R* undersampling factors, *n* times, and for *p* different perturbations. The reconstruction results are averaged over the realizations, and Fourier transformed along the frequency encode direction, since no undersampling is applied in this direction. Linear regression yields the maps **h**_*A*_ and **H**_*B*_ for calculation of the MTF and the signal-to-artefact maps. Finally, signal-to-artefact maps are used to mask the MTF results

Fully sampled and undersampled numerical phantoms were reconstructed using *k-t* PCA and *k-t* SPARSE-SENSE. Undersampling factors were 2, 5 and 10 excluding training data, corresponding to net factors of 1.9, 4.4, and 7.6 when including the central 11×7 training ellipse. Because of a steep decline of *k-*space magnitudes away from the centre, noise becomes dominant towards the edges of *k-*space. To mitigate this effect, 64 realizations of each set of simulations were done and the average reconstructed images were used for MTF analysis. The reconstructed images from undersampled data were compared to the fully sampled reference in *k-f* space using linear regression as detailed in eq. (8). MTFs and corresponding artefact measures were computed (cf. eqs. (9), (10)). To account for the drastic decrease of data magnitudes towards the *k-*space edges, MTFs were masked using thresholding on corresponding signal-to-artefact maps (S2A). The S2A threshold was empirically set to 3 for 3D MTF maps; a value which best separates parts of the MTF with low and high artefact proportion. The different steps employed for MTF analysis are illustrated in Fig. 2.

Timing constraints prohibit acquisition of a fully sampled 3D dataset during the first-pass of the contrast agent in-vivo. Hence, reference single slice 2D data were used for in-vivo MTF analysis. MTF calculations were performed using the same undersampling factors and procedure as for the MRXCAT phantom. In contrast to the MRXCAT case, training consisted of 11 profiles in *k*_*y*_ only, resulting in different net acceleration factors (1.9, 3.8, and 5.9), and the S2A threshold was set to 2.5.

### Image-time domain analysis

In addition to MTF analysis in *k-f* space, signal intensity vs. time curves extracted from MRXCAT images were investigated. Direct comparison of accelerated scanning simulations with fully sampled reference data allows for estimation of data fidelity upon undersampling during contrast enhancement. Furthermore, specific features of the signal intensity-time curve such as the pre-contrast baseline, peak enhancement and upslope can be compared. Errors in these features will directly propagate into the estimated myocardial blood flow upon signal-to-concentration conversion or deconvolution fitting.

### Myocardial blood flow quantification

Estimation of myocardial blood flow (MBF) was performed in two steps. First the image signal intensity vs. time curves from the blood pool and myocardium were converted to concentration vs. time curves using the signal model of the form [31]

13 $$ S={S}_0\cdotp \left(\left(1- \exp \left(-{R}_1\cdotp {T}_{\mathrm{SAT}}\right)\right)\cdotp {a}^{n-1}+\left(1- \exp \left(-{R}_1\cdotp {T}_R\right)\right)\cdotp \frac{1-{a}^{n-1}}{1- a}\right). $$

*S* represents the signal intensity, *T*_SAT_ the saturation delay, *T*_*R*_ the repetition time and *n* the number of profiles acquired between the acquisition start and the central *k-*space portion. *R*_1_ = 1/*T*_1_ is the dynamic relaxivity and the term *a* = cos *α* · exp(−*R*_1_ *T*_*R*_) additionally contains the flip angle *α*. The baseline time frames were used to determine the scaling factor *S*_0_ using pre-contrast *T*_1_ values. These values were either known for the MRXCAT simulations, or measured using MOLLI imaging for in-vivo data. Since *S*_0_ can be assumed unaffected by the Gadolinium administration, the dynamic *T*_1_ can be calculated with this *S*_0_ for each time frame. The relaxivity *R*_1_ is given by

14 $$ {R}_1=\frac{1}{T_1}=\frac{1}{T_{1,0}}+ c\cdotp r, $$

where *T*_1,0_ is the baseline *T*_1_ in the absence of contrast agent, and *r* the material-specific relaxivity of the contrast agent. Resolving eq. (14) yields the concentration *c* of the contrast agent.

Baseline ranges for signal-to-concentration conversion were set to time frames 1–5 for the AIF and 1–10 for the myocardial curves in all MRXCAT simulations. Since baseline length, timing of acquisition and contrast agent injection vary in-vivo, baseline range selection was done manually in each volunteer dataset. In-vivo population average pre-contrast *T*_1,0_ values derived from MOLLI imaging were: 1590 ms for the left ventricle and 1020 ms for the myocardium at the first contrast agent injection. *T*_1,0_ before the second injection were 640 ms and 680 ms, respectively.

In a second step, the concentration vs. time curves *c*_AIF_ and *c*_MYO_ from the blood pool and the myocardium, respectively, were related to estimate the MBF using Fermi model deconvolution as detailed in eqs. (11) and (12), and reference [27].

### Sub-endocardial Ischemic lesion simulation

The ability of the proposed 3D methods to reveal small ischemic defects was probed by MRXCAT simulation of sub-endocardial ischemia. Ischemia was introduced in a single slice of the MRXCAT phantom with a healthy rest MBF of 1 mL/g/min. The ischemic region in a mid-ventricular slice covered a circumferential lateral sector spanning 60°, and a transmural sub-endocardial layer of 1–2 voxels. In this ischemic territory, contrast enhancement was suppressed such that the signal intensities remained around the baseline level during all time frames. Ischemic MRXCAT data were reconstructed without undersampling and at 10x scan acceleration using both *k-t* PCA and *k-t* SPARSE-SENSE. Subsequently, MBF quantification was performed.

## Results

**Fig. 3:**
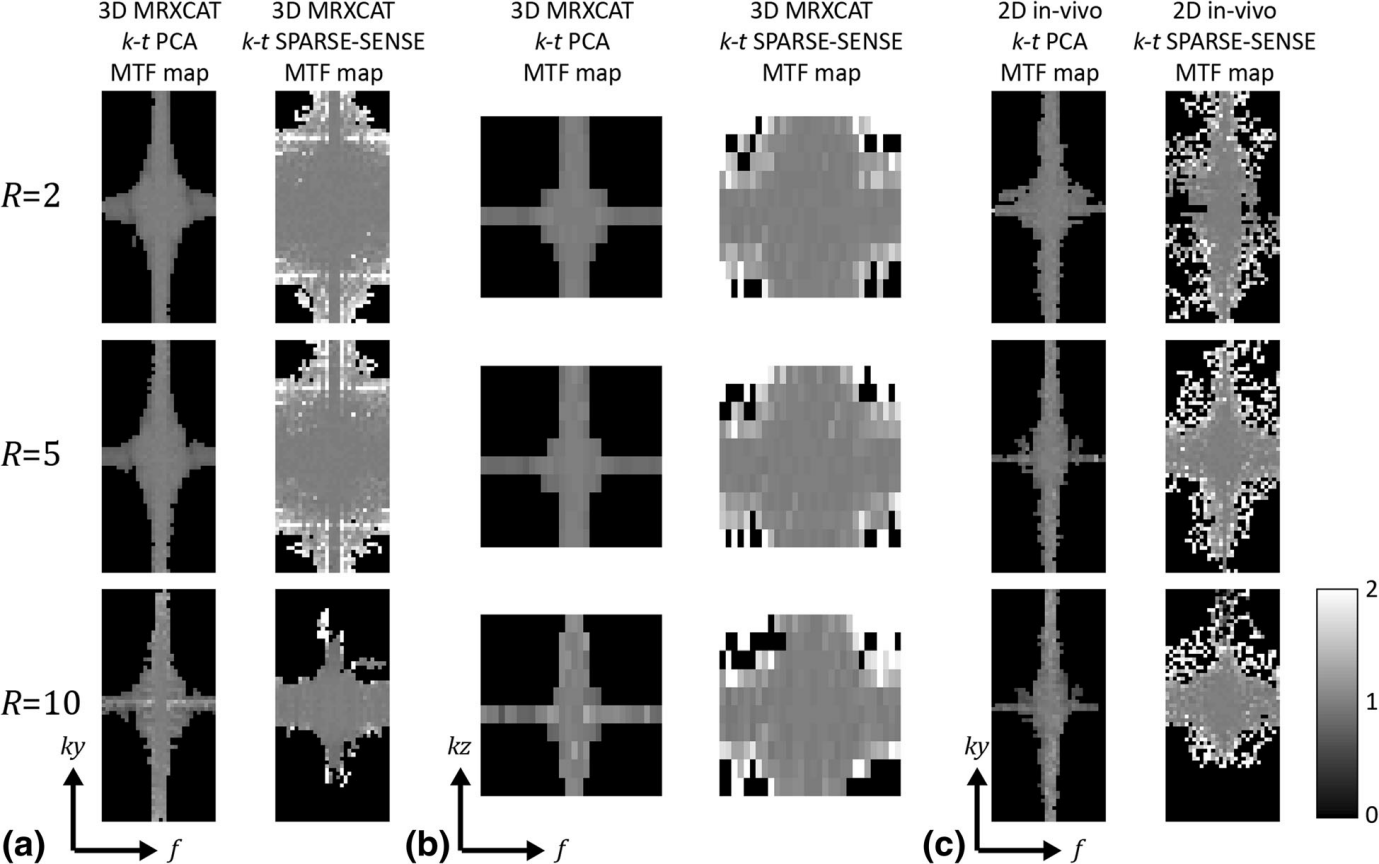
Modulation transfer function (MTF) simulation results comparing *k-t* PCA and *k-t* SPARSE-SENSE. MTFs for undersampling factors 2, 5 and 10 are shown in the *three rows*. **a**,**b** MTF maps using the MRXCAT numerical phantom plotted along *k*_*y*_-*f* at *k*_*z*_ = 0 (**a**), and along *k*_*z*_ *-f* at *k*_*y*_ = 0 (**b**). **c** MTF *k*_*y*_ *-f* maps derived from fully sampled 2D in-vivo data with retrospective undersampling. MTF maps were masked using an empirically determined threshold in the signal-to-artefact maps. Thresholds were set to 3 for 3D MRXCAT, and 2.5 for 2D in-vivo simulations

MTF simulation results are shown in Fig. 3. Thresholds in signal-to-artefact maps (S2A) were used to mask out regions with low SNR in MTF maps. 3D and 2D MTF maps were set to zero if the corresponding signal-to-artefact values were below 3 and 2.5, respectively. For 3D MRXCAT the MTF spans a 3D space in *k*_*y*_ *-k*_*z*_ *-f*.

Figure 3a displays a *k*_*y*_ *-f* slice of the MRXCAT MTF map at *k*_*z*_ = 0 for *k-t* PCA and *k-t* SPARSE-SENSE at nominal acceleration factors of 2, 5, and 10. A *k*_*z*_ *-f* slice at *k*_*y*_ = 0 of the MRXCAT MTFs is shown in Fig. 3b. For both reconstruction methods at all acceleration factors, the non-zero MTF values lie around the main axes, i.e. along the different direct current (DC) regions. In the temporal DC region, data at most spatial frequencies *k*_*y*_ and *k*_*z*_ are partially restored upon undersampling. Similarly, at spatial DC, all temporal frequency components are restored to a certain degree. MTF values decrease with increasing distance from the DC axes. For *k-t* PCA at different undersampling factors, the shape of the MTF remains similar with slight narrowing of the non-zero regions near the DC axes. At *R* = 10, the MTF is noisier than at lower acceleration indicating noise amplification at certain spatiotemporal frequencies. Compared to *k-t* SPARSE-SENSE, *k-t* PCA restores off-DC temporal frequencies on a relatively narrow range. As a consequence, MTFs from *k-t* SPARSE-SENSE have a larger non-zero area, but exhibit larger changes when increasing *R*. MTF values >1 away from the DC axes signify deviation from linear behaviour due to the non-linearity of the reconstruction algorithm. A number of spatial frequency components along *k*_*y*_ is not restored using 10x *k-t* SPARSE-SENSE. This leads to a loss of in-plane spatial resolution in the reconstructed image.

MTF results derived from 2D in-vivo data are illustrated in Fig. 3c, revealing similar patterns as for the 3D simulation along the DC axes. In contrast to 3D, 2D results exhibit lower signal-to-artefact ratios, yielding smaller non-zero MTF areas despite the slightly reduced signal-to-artefact threshold. As in the 3D simulation at maximum undersampling rate *R* = 10, *k-t* SPARSE-SENSE exhibits a loss of spatial resolution in phase-encoding direction.

**Fig. 4:**
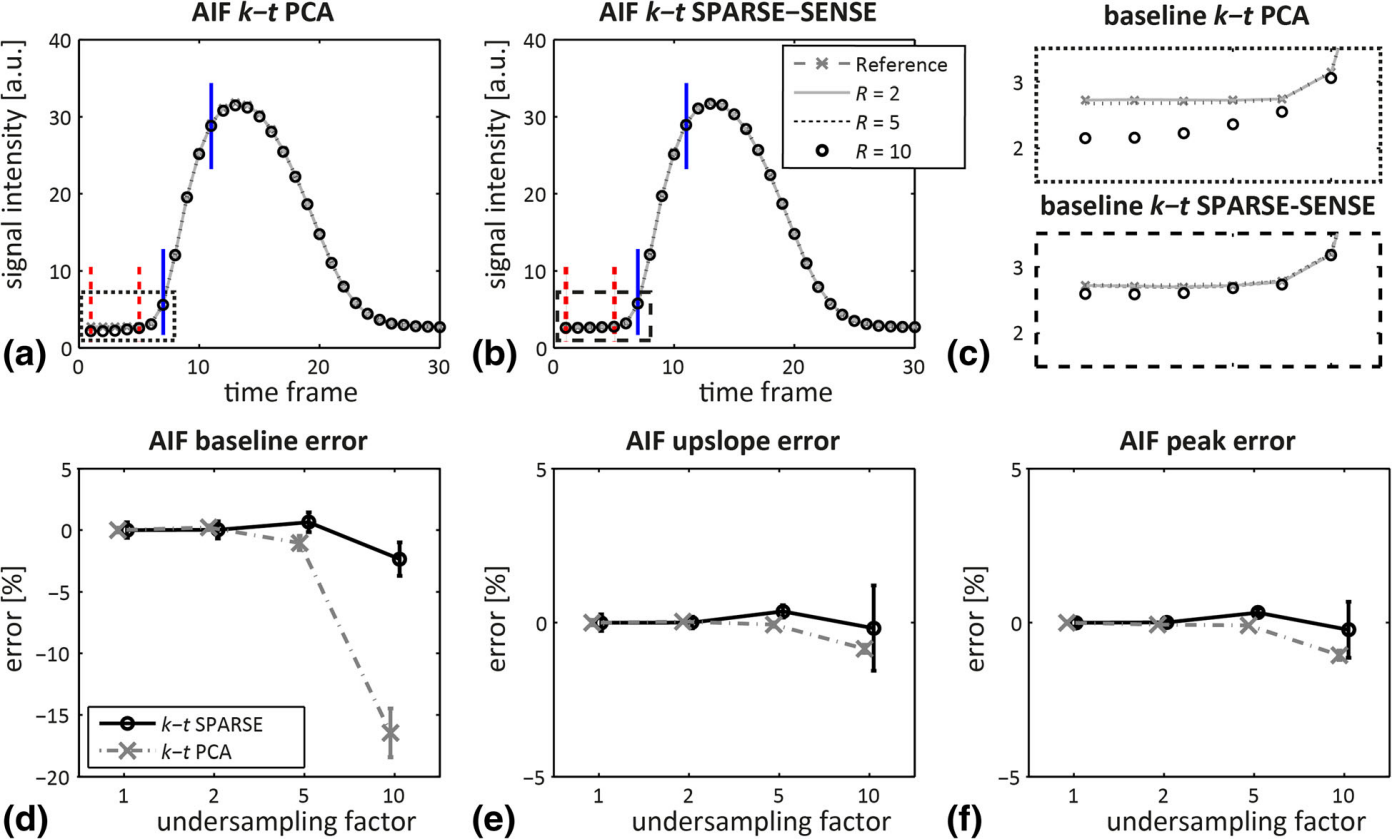
Arterial input functions (AIFs) derived from 3D MRXCAT simulations for different undersampling factors and signal intensity errors. AIFs from reconstructions using (**a**) *k-t* PCA and (**b**) *k-t* SPARSE-SENSE for fully sampled reference, and undersampling factors *R* = 2, 5, 10. Baseline (*dashed red*) and upslope limits (*solid blue*) are indicated in (**a**,**b**). **c** Zoom of the AIF baselines in (**a**), (**b**) as indicated by the dashed boxes. **d**-**f** Percentage error as a function of the undersampling factor in (**d**) baseline, (**e**) upslope and (**f**) peak signal. Error bars indicate mean and twice the standard deviation across 10 realizations of the simulation

AIFs extracted from central left-ventricular regions of the reconstructed 3D MRXCAT images for *R* = 1, 2, 5, 10 are presented in Fig. 4a,b. AIFs appear perfectly aligned for all *R* except for the baseline. A close-up of the baselines and corresponding error plot as a function of *R* reveals 16.5 ± 2.0% reduced baseline signal intensities at *R* = 10 for *k-t* PCA compared to the reference, while the baseline error is – 4.1 ± 1.4% for 10x *k-t* SPARSE-SENSE (Fig. 4c,d). Errors in the AIF upslope and maximum signal intensity are depicted in Fig. 4e,f, and remain below ±2% at all acceleration factors.

**Fig. 5:**
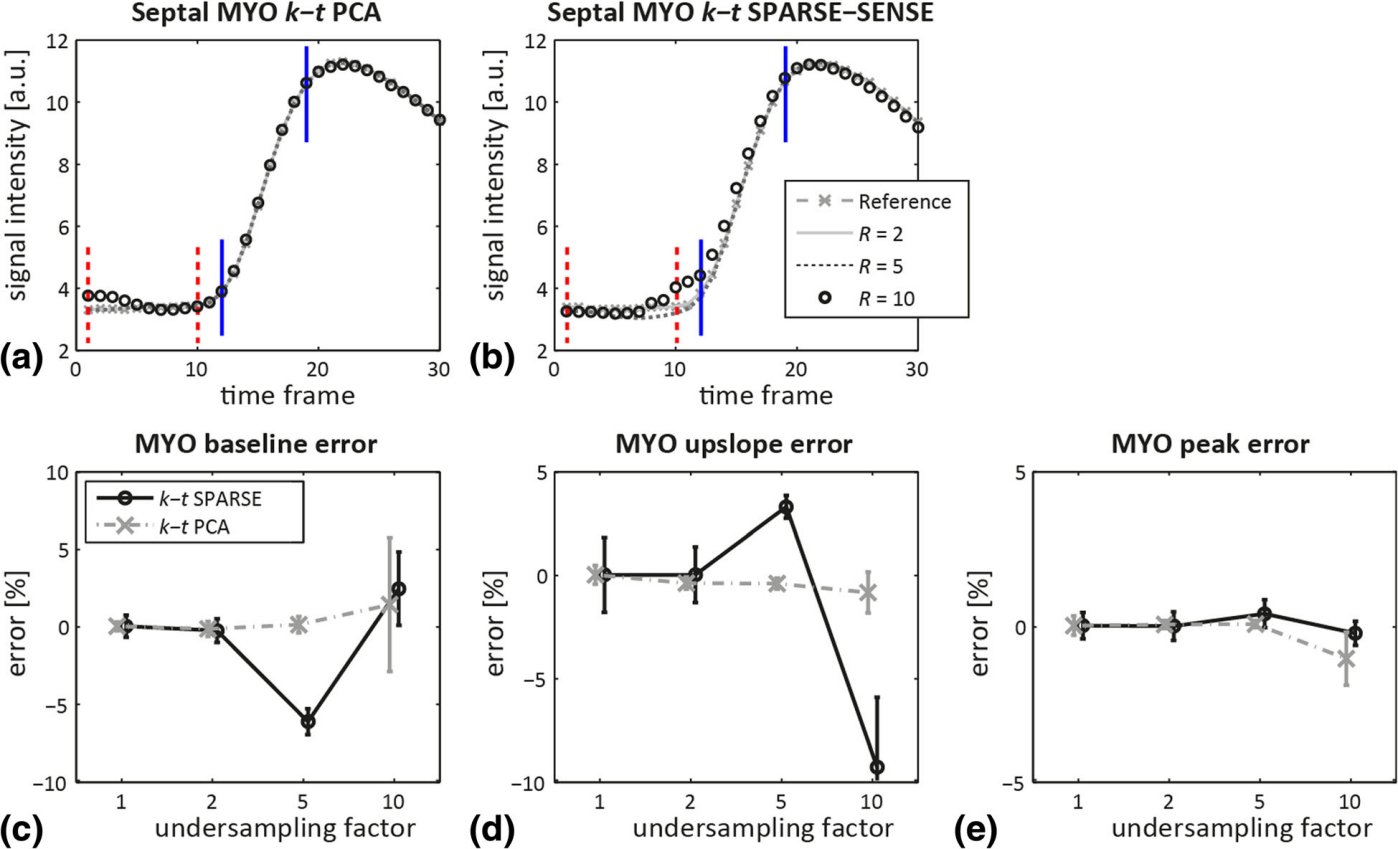
Myocardial (MYO) signal intensity vs. time derived from the septum in 3D MRXCAT simulations for different undersampling factors and signal intensity errors. Myocardial curves from reconstructions using (**a**) *k-t* PCA and (**b**) *k-t* SPARSE-SENSE for fully sampled reference, and undersampling factors *R* = 2, 5, 10. Baseline (*dashed red*) and upslope limits (*solid blue*) are indicated in (**a**,**b**). **c**-**e** Percentage errors as a function of the undersampling factor in (**c**) baseline, (**d**) upslope and (**e**) peak signal. *Error bars* indicate mean and twice the standard deviation across 10 realizations of the simulation

Figure 5 highlights myocardial signal intensity-time curves extracted from a septal segment at a mid-ventricular level of 3D MRXCAT simulations. Reference (*R* = 1) and *R* = 2, 5, 10 undersampled acquisitions are shown. Overall agreement between curves at all acceleration factors is good. Baseline errors are visible at *R* = 10 for both reconstruction methods, with increased signal intensity in the first time frames for *k-t* PCA, and an elevated baseline shortly before bolus arrival for *k-t* SPARSE-SENSE. As Fig. 5c reveals, these errors almost cancel out by averaging the baseline across the first 10 time frames. Mean baseline errors and standard deviations for 10 realizations of the simulations and 10-fold undersampling were 1.4 ± 4.3% for *k-t* PCA and 2.4 ± 2.4% for *k-t* SPARSE-SENSE. The myocardial upslope changes by – 0.9 ± 1.0% and – 9.4 ± 3.4% for 10x *k-t* PCA and SPARSE-SENSE, respectively. The peak signal intensity error stays below ±2% at all undersampling factors.

**Fig. 6:**
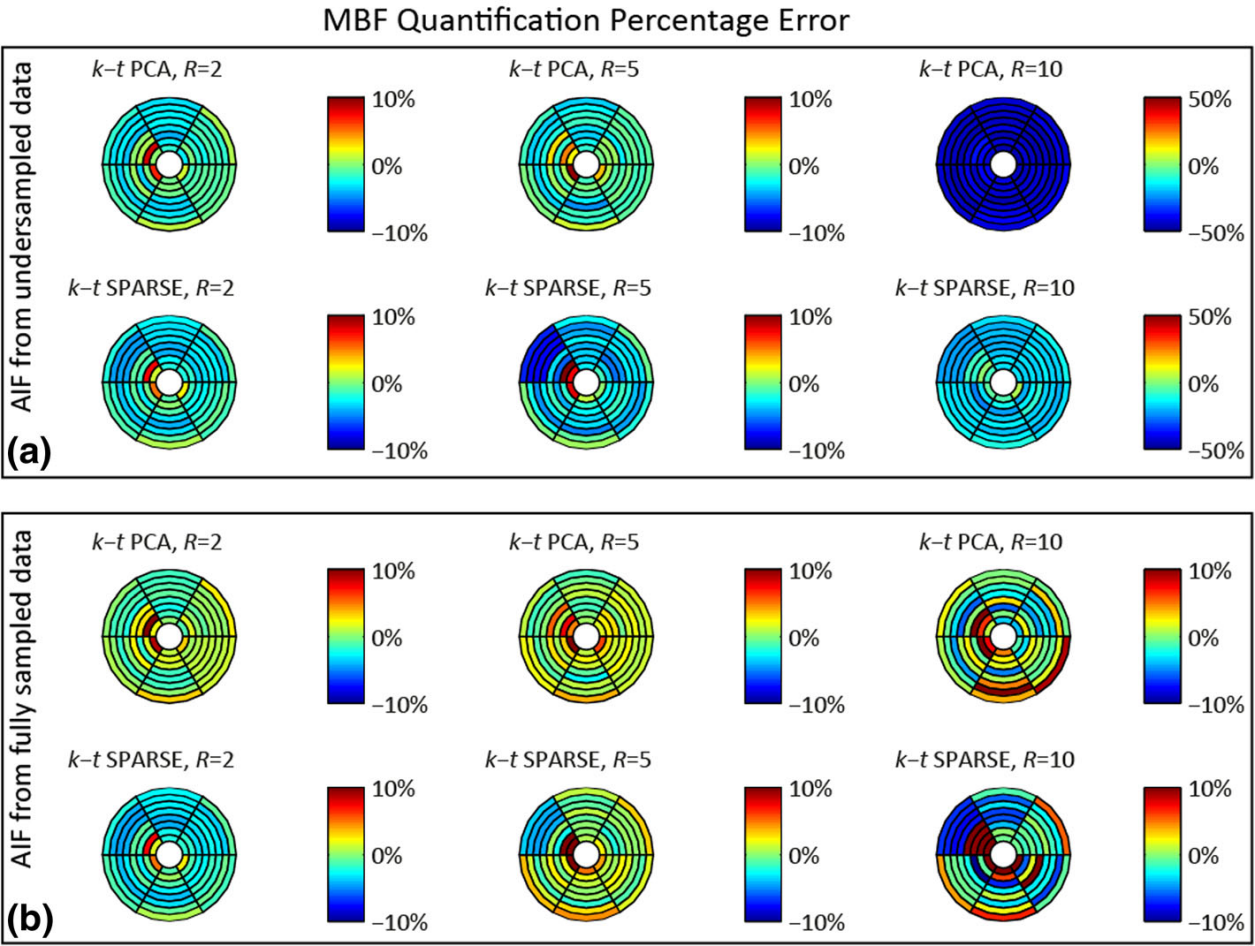
Percentage error upon MBF estimation for different realizations of the MRXCAT simulation. **a** Quantification errors in % for *k-t* PCA and *k-t* SPARSE-SENSE at undersampling rates *R =* 2, 5, 10 using AIF and myocardial curves extracted from the undersampled data. Note that colour axes for *R* = 10 were adjusted to portray the strong MBF underestimation. **b** Quantification errors [%] as in (**a**), derived using a reference AIF extracted from fully sampled data and myocardial curves from the undersampled data, as accomplished using dual-sequence acquisition. The strong MBF underestimation at *R* = 10 is reduced with the reference AIF

**Fig. 7:**
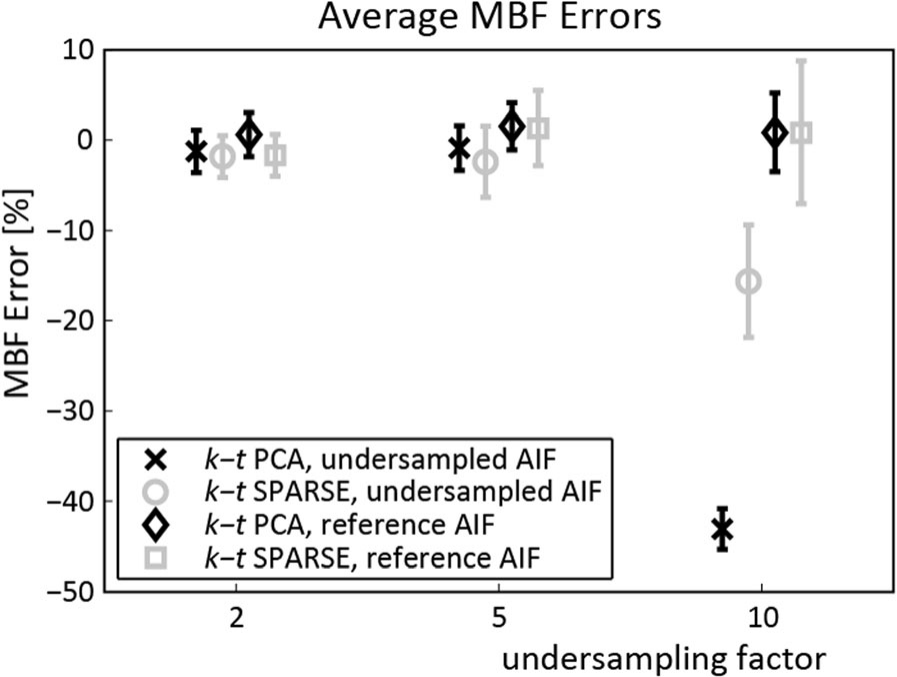
Average MBF errors and standard deviations in % across 48 myocardial regions (8 slices, 6 angular sectors) of the MRXCAT numerical simulation for undersampling factors *R =* 2, 5, 10 using *k-t* PCA and *k-t* SPARSE-SENSE. Strong MBF underestimation occurs upon quantification at *R* = 10 when the AIF and the myocardial signal intensity-time curves are extracted from the same image upon undersampling. The MBF errors are markedly reduced if the reference AIF is used. In this study, the reference AIF is extracted from a separate image acquired using interleaved scanning

Errors in estimated MBF were evaluated in 8 slices and 6 angular sectors of the 3D MRXCAT simulation with an AIF extracted from the undersampled image representing standard non-interleaved acquisition. In order to model interleaved scanning with separate AIF assessment, MBF quantification errors were also determined using an AIF derived from a fully sampled reference. Detailed results are depicted in the form of Bull’s eye plots in Fig. 6, and summarized in Fig. 7 as mean MBF errors and standard deviations across the 8×6 regions. Mean MBF errors remain below 3% for all evaluations at *R* = 2 and *R* = 5. In contrast, if the AIF is extracted from the undersampled data itself, MBF at 10x undersampling is underestimated by 43.1 ± 2.3% for *k-t* PCA, and 15.6 ± 6.2% for *k-t* SPARSE-SENSE. Underestimation is removed when the AIF from fully sampled data is employed for quantification, with average MBF errors of 0.8 ± 4.3% and 0.9 ± 7.9% for 10x *k-t* PCA and *k-t* SPARSE-SENSE, respectively. The variation of MBF errors across the myocardium rises alongside increasing the acceleration factor.

**Fig. 8:**
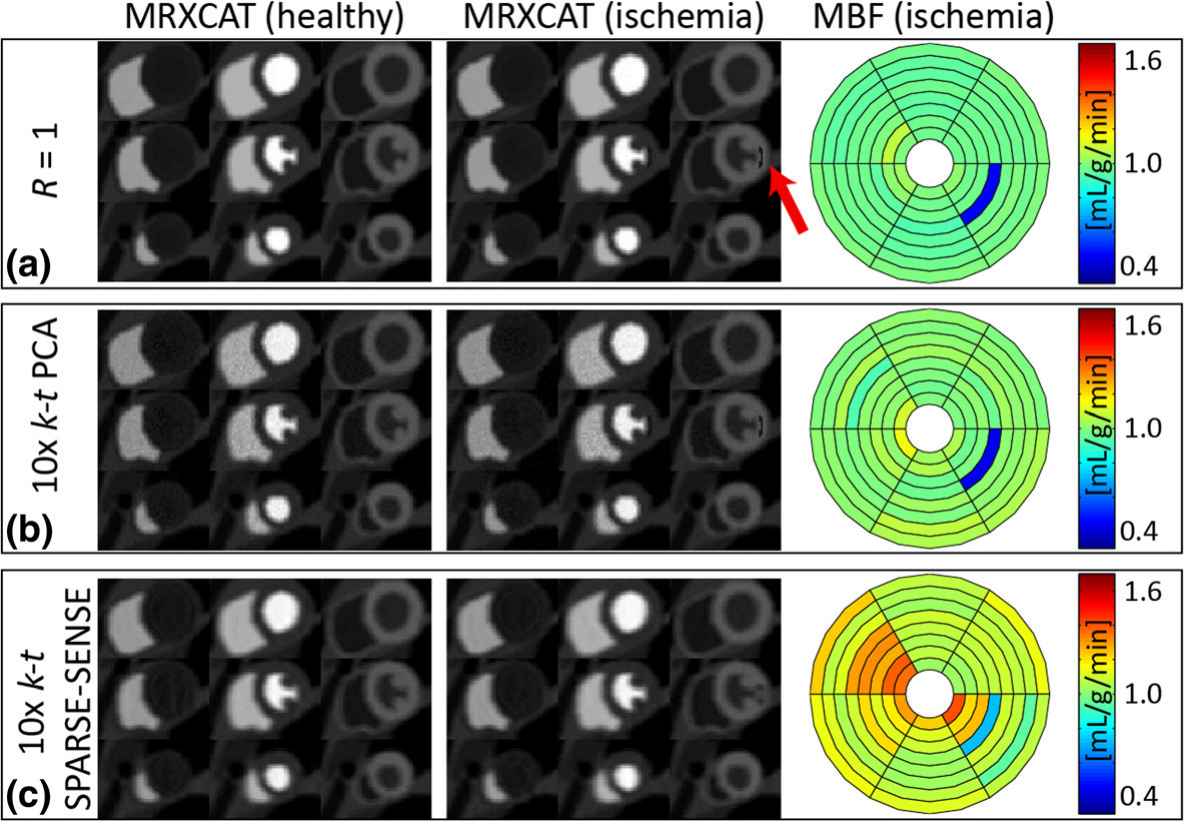
Example 3D MRXCAT simulation images for healthy and ischemic situations, and MBF quantification of the small sub-endocardial ischemia, for (**a**) full sampling, (**b**) 10-fold accelerated *k-t* PCA, (**c**) 10x *k-t* SPARSE-SENSE. 3 slices (basal, mid-ventricular, apical) at 3 time points of signal enhancement are shown. Sub-endocardial ischemia in one mid-ventricular slice was simulated (*red arrow*). Quantifications for *R* = 1 and 10x *k-t* PCA yield equally reduced MBF in the ischemic sector, while the healthy sectors remain unaffected. MBF in the ischemic sector is also lower in *k-t* SPARSE-SENSE, but MBF reduction is less pronounced

Figure 8 displays example MRXCAT images of healthy and diseased simulations. While in the healthy case dynamic contrast enhancement is homogeneous in all myocardial slices, a small sub-endocardial defect was introduced in the lateral segment of the mid-ventricular slice of the ischemia simulation. The ischemic lesion is very distinct in the fully sampled case and 10x *k-t* PCA, but less perceptible in 10x *k-t* SPARSE-SENSE. MBF estimation in the segment affected by ischemia yielded MBF = 0.46 mL/g/min for *R* = 1, 0.45 mL/g/min for 10x *k-t* PCA and 0.73 mL/g/min for 10x *k-t* SPARSE-SENSE. Due to the transmural averaging of myocardial signal including sub-endocardial ischemic and epicardial healthy voxels, the resulting MBF is larger than zero.

**Fig. 9:**
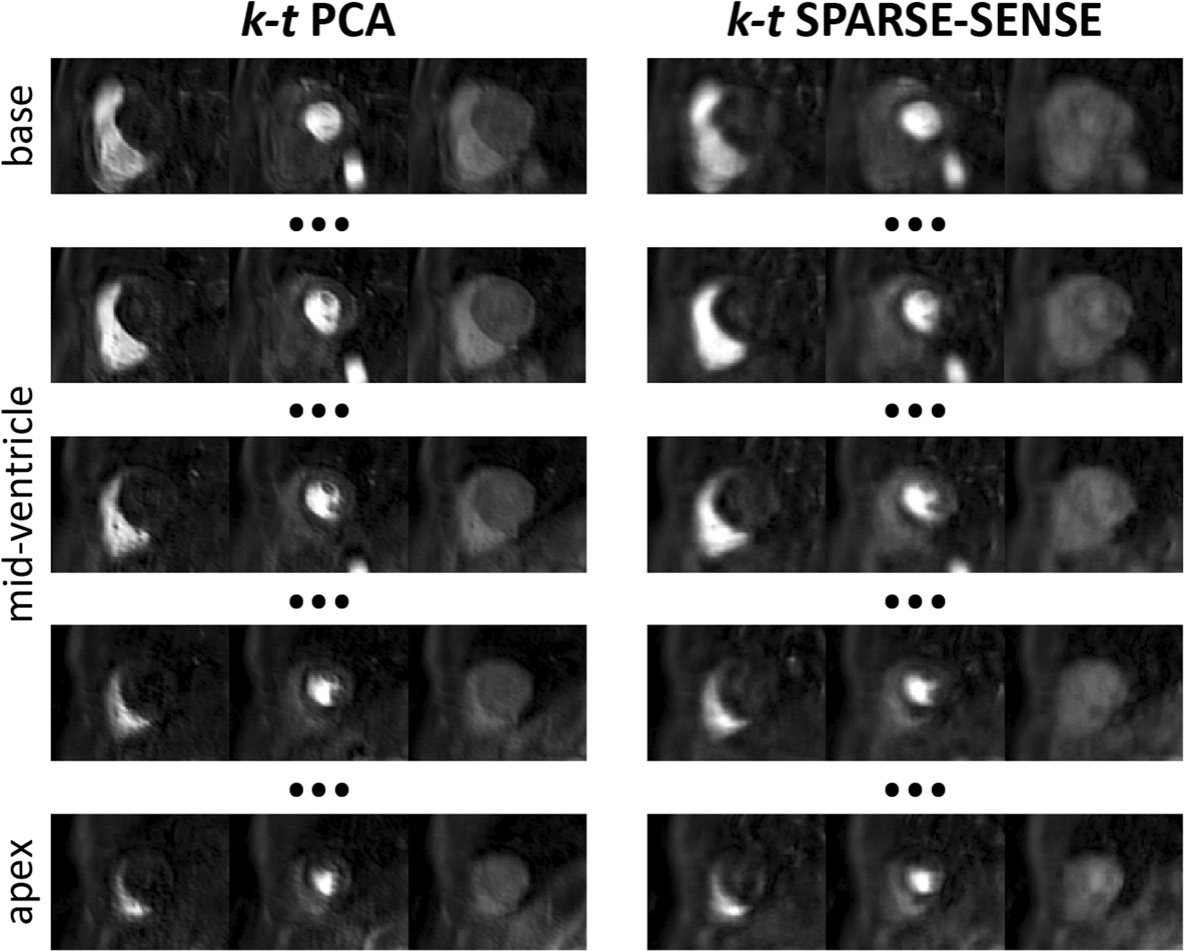
Example short-axis slices of in-vivo images using 10-fold accelerated *k-t* PCA and 10x *k-t* SPARSE-SENSE perfusion imaging in one volunteer. Time frames of peak contrast enhancement in the right ventricle, left ventricle and myocardium are shown in 5 slices from base to apex (gap of 1 slice between the shown slices)

In-vivo images comparing 10x accelerated *k-t* PCA and *k-t* SPARSE-SENSE are illustrated in Fig. 9. Five different slices from apex to base are displayed at time points of maximum contrast enhancement in the right ventricle, left ventricle, and myocardium. One slice was omitted in-between slices thereby spanning nine slices. Both images display similar contrast enhancement, but while *k-t* PCA images display sharp tissue boundaries, *k-t* SPARSE-SENSE images appear more blurred.

**Fig. 10:**
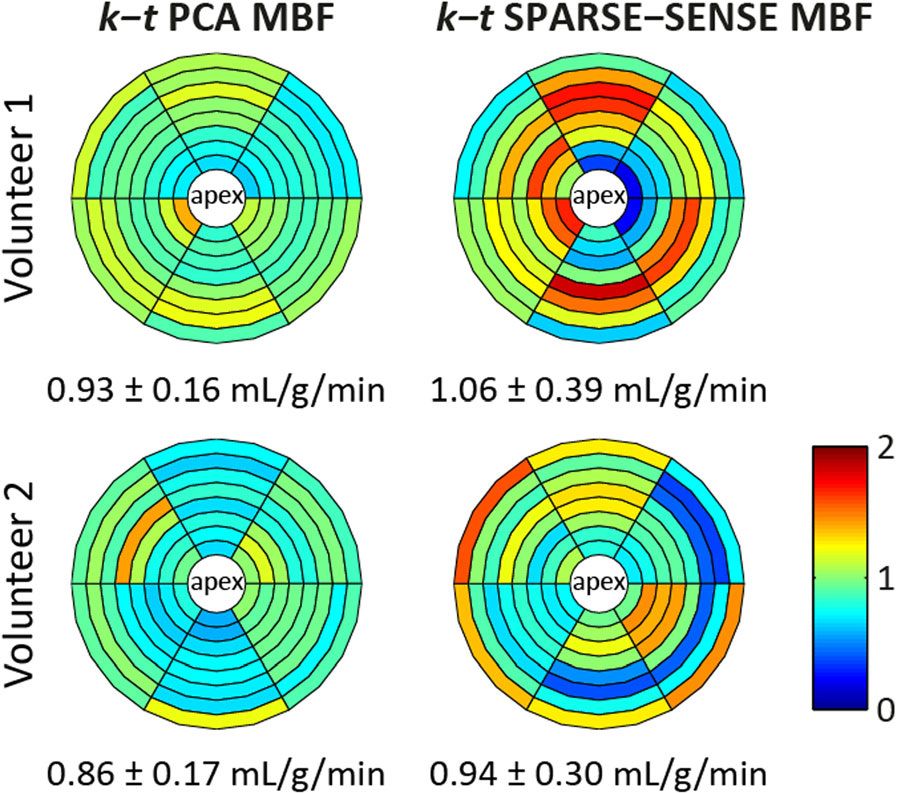
Example in-vivo MBF estimation results comparing *k-t* PCA and *k-t* SPARSE-SENSE in two volunteers. Mean MBFs and standard deviations across different myocardial regions are lower for *k-t* PCA than for *k-t* SPARSE-SENSE

Figure 10 shows example MBF estimates derived from in-vivo 3D *k-t* PCA and *k-t* SPARSE-SENSE images acquired with 10x undersampling. Average MBF values across 8 slices and 6 sectors per slice in the first volunteer were 0.93 ± 0.16 mL/g/min for *k-t* PCA and 1.06 ± 0.39 mL/g/min for *k-t* SPARSE-SENSE. For the second volunteer shown, mean MBF and standard deviations amounted to 0.86 ± 0.17 mL/g/min and 0.94 ± 0.30 mL/g/min, respectively. The larger standard deviations of MBF in *k-t* SPARSE-SENSE appear as increased inhomogeneity in MBF values across the Bull’s eye plots.

**Fig. 11:**
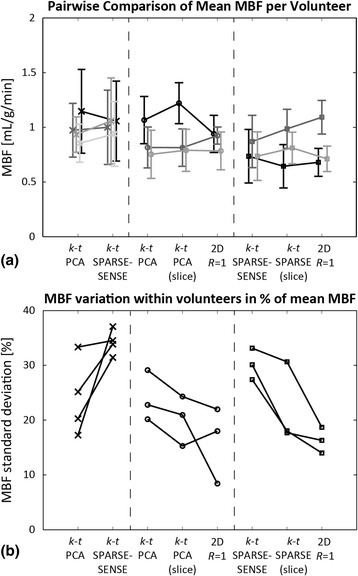
Summary of in-vivo myocardial blood flow (MBF) values. **a** Grouped comparison of mean MBF ± standard deviation and (**b**) intra-volunteer variation across sectors within 10 volunteers. Two out of the three acquisition types (10× 3D *k-t* PCA, 10× 3D *k-t* SPARSE-SENSE, 2D *R* = 1) were performed in each volunteer. For 3D vs. 2D comparisons, a mid-ventricular region of the 3D volume corresponding to the 2D imaging slice was additionally evaluated, indicated by (slice). Intra-volunteer MBF variation was lower in fully sampled cases than in accelerated CMR, and lower in *k-t* PCA than in *k-t* SPARSE-SENSE

A summary of average MBF and standard deviations for all volunteers is provided in Fig. 11. Volunteers were grouped according to the first-pass perfusion techniques to enable side by side comparison between acquisition methods within volunteers. In addition to whole-heart evaluation of 3D images, quantification was also performed in a mid-ventricular region consisting of two averaged slices of the 3D images. The averaged region with effective slice thickness of 10 mm corresponded to the 2D imaging region. This step was done to increase comparability between 3D *R* = 10 and 2D MBF values. Average MBFs ranged from 0.64 and 1.22 mL/g/min and agreed well between methods. Ratios between mean *k-t* PCA and *k-t* SPARSE-SENSE MBF ranged from 0.88 to 1.08. Comparison between accelerated *k-t* and 2D *R* = 1 methods yielded factors of 0.88 to 1.30 for *k-t* PCA and 0.90 to 1.14 for *k-t* SPARSE-SENSE. MBF standard deviations within volunteers normalized to the corresponding mean MBF were 16.3 ± 4.7% for 2D, 25.2 ± 5.5% for 10x *k-t* PCA, and 32.5 ± 3.2% for 10x *k-t* SPARSE-SENSE. MBF standard deviations were higher in accelerated 3D scans than in fully sampled 2D images. Comparison of the two accelerated methods yielded lower MBF variation in *k-t* PCA than *k-t* SPARSE-SENSE.

## Discussion

The feasibility of MBF estimation from highly undersampled first-pass myocardial perfusion MRI has been investigated and presented in this work. Effects were examined by means of *k-f* space based MTFs, image-time domain analysis of signal intensity, and by deconvolution using Fermi function modelling for MBF estimation. The MRXCAT framework [53] was employed for simulation, and complemented by in-vivo assessment of perfusion using accelerated 3D *k-t* PCA, 3D *k-t* SPARSE-SENSE and fully sampled 2D reference data.

The concept of the MTF describing the relationship between an imaged object and its image has been adapted to portray undersampled first-pass perfusion CMR. Thereby, the MTF represents the relationship in *k-f* space between the fully sampled and the undersampled data upon image reconstruction. Implementation in MRXCAT allowed for quantification of errors relating the accelerated imaging simulation to the corresponding fully sampled reference. The reduction in MTF area with increasing acceleration factor and the appearance of noise therein provide insights into the performance of the undersampling and reconstruction strategy.

For *k-t* PCA, the *k*_*y*_ *-f* portion with MTF close to 1 remains almost unchanged from *R* = 2 up to *R* = 10, suggesting adequate performance of image reconstruction at all examined *R*. The increased noise-like patterns in 10x *k-t* PCA MTFs indicate that this acceleration factor is close to the maximum achievable *R* without major loss of data fidelity. On the other hand, *k-t* SPARSE-SENSE MTFs exhibit larger non-zero areas for all temporal frequencies further away from the spatial DC. MTF shapes at *R* = 2 and *R* = 5 are similar, but a sudden drop-off at high *k*_*y*_ is observed at *R* = 10, indicating that data at higher spatial frequencies are not properly restored. This yields a loss in effective spatial resolution, which can be observed in-vivo comparing *k-t* PCA and *k-t* SPARSE-SENSE images in Fig. 9.

Starting from the original definition of the MTF as a relationship between the object and its image, the MTF may assume values between 0 and 1 because information about the object is only lost and never gained with bandlimited, linear imaging methods. However, when applied to the characterization of undersampling, MTF > 1 is possible indicating noise amplification at the corresponding *k-f* position by regularized reconstruction. This phenomenon is most prominent at high undersampling rates, e.g. in the *k*_*y*_ *-f* MTF map for 10x *k-t* PCA. Around spatial DC, some temporal frequencies exceed 1 resulting in the noise-like MTF appearance. In contrast, MTF values vastly exceeding 1, as observed only in *k-t* SPARSE-SENSE MTF maps at higher *k*_*y*_, may not be explained by noise amplification alone. Presumably, these errors stem from the treatment of non-linear compressed sensing reconstruction with the linear MTF formalism. The assumption of linearity between images from fully sampled and undersampled acquisitions is violated at higher frequencies, which is in line with previous statements [41]. The discrepancy between linear and non-linear reconstruction algorithms treated with the MTF formalism was corrected for using masking with a fixed threshold in the signal-to-artefact map.

Simulated signal intensity-time curves from the blood pool and the myocardium were examined at all acceleration factors *R*. The AIFs for different *R* agree well when upslopes and peak signal are compared, as well as for the baseline up to *R* = 5. Underestimation of the baseline at *R* = 10 is most prominent in *k-t* PCA with almost 20% error. The myocardial curves up to *R* = 5 agree well with the reference, but exhibit deviations from ground truth at the beginning (*k-t* PCA) or at the end of the baseline (*k-t* SPARSE-SENSE). These errors are reflected in the myocardial baseline error, which can be reduced if the time frames selected for baseline averaging are optimally chosen. Based on these findings, the first time frames might be excluded when determining the baseline in *k-t* PCA. Accordingly, for *k-t* SPARSE-SENSE, the last time points before contrast agent arrival should be discarded. The septum was chosen for myocardial signal-time analysis due to its strategic position between the right and left ventricle. Aliasing of components from left and right ventricles and the myocardium is expected in the septum upon undersampling, as these three compartments are aligned along the fold-over direction. Resolving the aliased data at this location should be more challenging than anywhere else in the myocardium [17].

The percentage errors upon MBF quantification using AIFs extracted from the undersampled image and from a fully sampled reference were compared. Global MBF underestimation up to 43% was observed at *R* = 10 with the AIF from undersampled data, an error not present when using the AIF from reference image. This finding indicates that the AIF baseline error may be the main source of inaccuracy. A remedy to address this issue in-vivo is interleaved AIF acquisition at small acceleration factors using dual-sequence imaging, thereby markedly reducing the AIF baseline error. Exact knowledge of sequence parameters included in the corresponding signal model was assumed in this simulation, alongside with perfect saturation efficiency. As previously shown, errors in parameter estimation as well as inefficient saturation may additionally distort the estimated MBF [54]. In addition, signal intensity to concentration non-linearity effects may further degrade quantification accuracy for single-sequence acquisition schemes.

Identification of sub-endocardial ischemia is a key criterion for the clinical utility of novel myocardial perfusion scan and post-processing methodology. MRXCAT simulations of fully sampled and 10x accelerated imaging including a small ischemic lesion were performed to investigate this question. Quantification of 10x *k-t* PCA data yielded MBF values in good agreement with the fully sampled reference both in healthy and ischemic regions. In contrast, MBF values derived from the 10x *k-t* SPARSE-SENSE differed from the reference in healthy segments, with increased MBF variation. In the ischemic territory MBF reduction due to ischemia was less pronounced than in the reference. This latter effect may be related to the loss of effective spatial resolution observed in 10x *k-t* SPARSE-SENSE MTF analysis.

In-vivo data were measured using a dual-sequence acquisition framework enabling separate images mapping blood pool and myocardial enhancement [27]. For 3x *k-t* PCA the AIF baseline error remained below 2% as confirmed by our simulations up to *R* = 5. In addition, dual-sequence imaging enabled separately optimized saturation delays for the interleaved scans, thereby eliminating the signal vs. concentration non-linearity concerns.

The range of average MBF values found in-vivo at rest was in line with previous findings. Variations of MBF across different volunteers are expected based on physiological differences. The change in mean MBF between different acquisition techniques is lower than the intra-volunteer MBF variation, and standard deviations in MBF around 20% compare well to previous work. This variation represents a persistent limitation of MBF quantification in part caused by the ill-posed nature of deconvolution fitting [49]. The increased intra-volunteer variation observed in highly accelerated vs. fully sampled reference data can be explained in part by the loss in data fidelity and SNR caused by undersampling. To enhance MBF estimation precision, increasing the contrast-to-noise ratio by high dose first-pass imaging is an option [27]. Furthermore, parallel imaging with up to 32 receive channels has been demonstrated to enhance image quality [55]. Moreover, in accelerated first-pass perfusion CMR accurate segmentation of the myocardium is crucial. For instance, the sector-wise myocardial signal intensity-time curve in the septum may be severely distorted if a single voxel from the right ventricle or multiple voxels affected by partial volume effects are included in the segmentation. These challenges need to be addressed in order to adopt fully quantitative perfusion CMR in clinical routine.

In addition to solving the aforementioned implementation challenges, further validation is needed before clinical introduction of the proposed methods. Future studies could include patients with sub-endocardial ischemia to investigate the ability to detect small, localized lesions. In addition, patients with triple vessel disease or microvascular disease potentially benefit from quantitative methods and may be included in clinical studies. In these pathologies, healthy remote myocardium may be absent as a reference for qualitative or semi-quantitative approaches.

## Conclusion

Combined modulation transfer function and signal-to-artefact ratio analysis is a useful means of studying the performance of accelerated 3D first-pass perfusion CMR acquisition in a linearized regime, correctly predicting losses in spatial and temporal resolution. Highly accelerated perfusion CMR enables estimation of myocardial blood flow provided an unbiased arterial input function is acquired, e.g. using dual-sequence acquisition. The accuracy of blood flow quantification from undersampled imaging is maintained compared to fully sampled reference images, whereas the precision measured by intra-volunteer variation is reduced prompting for further improvements of whole-heart 3D perfusion imaging approaches.

## Abbreviations

AIF: Arterial input function
CAD: Coronary artery disease
CMR: Cardiovascular magnetic resonance
DC: Direct current
FT: Fourier transform
MBF: Myocardial blood flow
MTF: Modulation transfer function
PC: Principal component
PCA: Principal component analysis
PET: Positron emission tomography
S2A: Signal-to-artefact map
SNR: Signal-to-noise ratio
SPECT: Single photon emission computed tomography
